# The Critical Interspecies Transmission Barrier at the Animal–Human Interface

**DOI:** 10.3390/tropicalmed4020072

**Published:** 2019-04-25

**Authors:** Kanta Subbarao

**Affiliations:** WHO Collaborating Centre for Reference and Research on Influenza and Department of Microbiology and Immunology, University of Melbourne, Peter Doherty Institute for Infection and Immunity, 792 Elizabeth Street, Melbourne, VIC 3000, Australia; kanta.subbarao@influenzacentre.org; Tel.: +03-9342-9310

**Keywords:** influenza, species barrier, cross-species infection, zoonoses, animal–human interface

## Abstract

Influenza A viruses (IAVs) infect humans and a wide range of animal species in nature, and waterfowl and shorebirds are their reservoir hosts. Of the 18 haemagglutinin (HA) and 11 neuraminidase (NA) subtypes of IAV, 16 HA and 9 NA subtypes infect aquatic birds. However, among the diverse pool of IAVs in nature, only a limited number of animal IAVs cross the species barrier to infect humans and a small subset of those have spread efficiently from person to person to cause an influenza pandemic. The ability to infect a different species, replicate in the new host and transmit are three distinct steps in this process. Viral and host factors that are critical determinants of the ability of an avian IAV to infect and spread in humans are discussed.

## 1. Introduction

Much of what we know about pandemic influenza viruses is based on analysis of the four influenza A viruses (IAVs) that caused influenza pandemics in the last 100 years: the 1918 H1N1, 1957 H2N2, 1968 H3N2 and 2009 H1N1pdm09 viruses. Animal hosts, primarily avian species and swine are the source of novel IAVs that are introduced into the human population. Over the last 20 years, hundreds of sporadic human infections caused by a range of different avian IAV subtypes and a few swine virus subtypes have been identified. This review examines the factors that affect the ability of IAVs to cross the species barrier into humans

## 2. Natural Reservoirs and Animal Hosts

Influenza A viruses infect many different species in nature, including water fowl and shorebirds, wild birds, chicken, turkeys, pigs, horses, dogs, cats, bats and humans [1]. To date, 18 haemagglutinin (HA) and 11 neuraminidase (NA) subtypes of IAVs have been described and, with modern virus discovery methods, it is likely that additional subtypes will be identified [2,3,4]. Waterfowl and shorebirds are believed to be the natural reservoir of IAVs, because (i) viruses of many subtypes (16 HA and 9 NA subtypes) have been isolated from them, (ii) infection is usually asymptomatic and (iii) viruses are generally in ecological stasis in these species [5]. In contrast to avian hosts, other species including pigs, horses and humans are infected by relatively few IAV subtypes. For example, classical swine influenza (which refers to H1N1 virus infection in pigs), H1N1pdm09 and H3 viruses infect pigs, H3 and H7 subtypes have caused equine influenza and sustained epidemics in humans have only been caused by H1, H2 and H3 IAV. 

The IAV genome is comprised of eight gene segments of negative-sense, single-stranded RNA. The enormous genetic diversity of influenza viruses in nature is a result of an error-prone RNA-dependent RNA polymerase that lacks proof-reading function, and genetic reassortment, whereby progeny viruses can acquire one or more novel gene segments when a host cell is co-infected by two different IAVs. These processes lead to antigenic drift and antigenic shift in IAVs, both of which occur continuously in nature.

## 3. Host-Range Determinants: Attachment, Infection and Replication

The host-range of an IAV is determined by the ability of the virus to infect and replicate productively in a host; critical host-range determinants are present in both the virus and the host. 

The viral haemagglutinin (HA) plays several important roles in IAV infection. First, it is the attachment protein of the virus and secondly, it has to be cleaved by tissue proteases for infectivity. The HA binds to its receptor, sialic acid (SA), on the surface of the host cell to initiate viral entry. The HAs of avian IAVs preferentially bind SAs linked to galactose by an α2,3 linkage while HAs of human IAVs preferentially bind α2,6-linked SA [6]. These observations have been further refined in the past decade; human IAVs bind long-chain α2,6-linked SA in an umbrella-shaped topology while avian IAVs bind α2,3-linked SA in a cone-shaped topology [7]. The receptor specificity of IAVs is assessed by lectin binding or by inference from specific residues (amino acids 226 and 228 in H3 and 190 and 225 in H1) in the predicted amino acid sequence of the viral HA [8]. 

The relevant SA receptors are present on epithelial cells lining the respiratory tract of humans and the gastrointestinal tract of birds. It is generally stated that α2,6 SA receptors are present in the upper respiratory tract of humans and α2,3 SA receptors are present in the lower respiratory tract, distal to the respiratory bronchioles [9,10]. However, this is an oversimplification; a careful characterization of the N- and O-glycan composition of the human lung, bronchus and nasopharynx by mass spectrometry revealed that there was a wide spectrum of α2,3 and α2,6 glycans in the lung and bronchus [11]. Furthermore, when these data were combined with binding data from commonly used glycan arrays, even the most comprehensive array that contained the greatest diversity of sialylated glycans, was not predictive of productive replication in the bronchus and lung [11]. Therefore, inferences about the receptor specificity of novel IAVs and predictions about their host range should be made with caution. 

The viral HA is synthesized as a polyprotein called HA0 that is cleaved into HA1 and HA2 domains at a conserved arginine residue; this cleavage is required for infectivity. The HAs of human and most avian IAVs are cleaved by trypsin-like proteases that are present in the respiratory tract of humans and GI tract of birds and this determines the tissue tropism of IAVs in these species. Some H5 and H7 avian IAVs acquire a motif of several basic amino acids in the HA cleavage site (multibasic cleavage site, MBCS), resulting in a HA that can be cleaved by non-trypsin like proteases and furins that are present in a much wider range of tissues [12,13]. Viruses bearing highly cleavable HAs cause a rapidly fatal, multi-system infection in poultry that is called highly pathogenic avian influenza (HPAI). Avian IAVs that lack the MCBS are referred to as low pathogenicity avian influenza (LPAI) viruses [14]. 

Highly pathogenic avian H5N1, H7N7 and, more recently, H5N6 and H7N9 IAVs that emerged in poultry have caused human infections in several countries raising concerns about their pandemic potential. However, it should be noted that the HPAI human infections have not spread from person to person and none of the pandemic viruses from the last century were derived from HPAI viruses.

The virally encoded polymerase complex, a heterotrimer of three polymerase proteins (PB2, PB1 and PA) is responsible for the replication of the viral genome. Key host-range determinants have been identified in the PB2 protein, notably at amino acids 627 and 701 in PB2 in avian origin viruses [15,16] or residues 590/591 in H1N1pdm09 [17]. It is assumed that there are critical interacting proteins in different species that bind or inhibit the corresponding polymerase proteins [18]. The chicken ANP32 protein [19] and importin-α proteins that are involved in nuclear transport of many cellular proteins are examples of host factors that play an important role in determining species specificity of IAVs [20]. Furthermore, erroneous polymerase activity during viral RNA replication of some influenza viruses can lead to the production of short aberrant RNAs, referred to as mini viral RNAs that can act as innate immune agonists and trigger cytokine dysregulation [21]. 

## 4. Infections across the Species Barrier

Although mammals are generally infected by a limited range of IAV subtypes, it is clear that interspecies transmission of IAVs is more common than was previously thought. Sporadic human infections by a range of avian IAVs (H4, H5, H6, H7, H9 and H10) and swine IAVs (H1 and H3) have been reported over the last 20 years [1]. Many of these infections are associated with severe disease and a high case fatality rate (H5, H7, H10), while others have been associated with mild, self-limited illness (H3N2v, H6, H9). Notably, very few of these infections have spread from person to person. Conversely, human IAVs have infected and become established in pigs [22], so influenza in swine today is caused by a variety of IAVs including classical swine (H1N1), human H3N2, H1N1pdm09 and reassortants among them [23]. Equine H3N8 and avian H3N2 viruses have transmitted to and became established in dogs [24,25,26].

## 5. The Feared Consequence of Interspecies Transmission

Influenza pandemics occur when a novel influenza A virus, to which a significant proportion of the human population lacks immunity, infects humans and is able to spread efficiently from person to person through the airborne route, causing sustained chains of community-wide transmission. The introduction and spread of antigenically novel IAVs in humans is referred to as antigenic shift. Novel influenza viruses are introduced from an animal reservoir. If the virus is able to spread efficiently and if a large enough proportion of the population is susceptible to infection, the novel virus can spread around the world in a pandemic and usually replaces the previously circulating virus. 

## 6. The Origins of the 1918, 1957, 1968 and 2009 Pandemic Viruses

The source and origin of the IAVs that caused the last four pandemics have been researched extensively in an effort to understand which IAVs pose a pandemic threat. 

The 1957 H2N2 and 1968 H3N2 pandemic influenza viruses were genetic reassortants that derived three [HA, NA and polymerase basic protein 1 (PB1)] or two (HA and PB1) gene segments, respectively, from an avian influenza virus and the remaining gene segments from the previously circulating human influenza virus [27,28]. The host in which these reassortment events occurred have not been identified. In principle, for reassortment to occur, the putative host would have to be productively coinfected with avian and human IAVs. Pigs were proposed to serve as the ‘mixing vessel’ because they support replication of IAVs from both species [29]. 

In contrast, the 1918 H1N1 virus is believed to have derived all of its genes from an avian IAV, though this has been debated in the literature [30,31,32]. Pigs and humans were infected by the 1918 virus around the same time, though the virus evolved differently in the two species. Phylogenetic analysis demonstrated that the 2009 H1N1pdm09 virus was derived from a reassortment of a North American lineage swine virus and a Eurasian avian-like virus circulating in pigs. It is not known when, where and in which pigs this reassortment event occurred. Since 2009, the virus has been isolated from pigs in many countries.

## 7. Sporadic Human Infections with Avian IAVs

In addition to the four viruses that caused pandemics, several avian IAVs have crossed the species barrier to infect humans but have not caused a pandemic, including hundreds of cases of H5N1 and H7N9 infection and sporadic cases of H5N6, H6N1, H9N2, H7N7, H7N3, H7N4, and H10N8 infection [33]. All of these infections were caused by wholly avian IAVs and almost all cases were associated with exposure to infected poultry. Compared to the four viruses that caused influenza pandemics, with very rare exceptions in close contacts, these AIVs were not associated with secondary spread. Thus, although they crossed the species barrier, and the human population lacks immunity to these subtypes, they failed to spread from person to person.

## 8. Transmissibility of Influenza Viruses that Cross the Species Barrier

The molecular determinants of the transmissibility of influenza viruses are not well understood. Epidemiologically successful IAVs, e.g., seasonal and pandemic influenza viruses, transmit efficiently from person to person while those that fail to do so cause sporadic infections. Ferrets and guinea pigs are the two animal models that are most widely used to evaluate the transmissibility of IAVs. Typically, the ability of IAVs to be transmitted to a naïve cage-mate (contact transmission) and to a naïve animal in an adjacent cage separated from physical contact with the infected animal by a mesh (airborne transmission) are assessed. While seasonal influenza A viruses transmit by contact and airborne transmission, avian H5N1 and H9N2 viruses fail to transmit by the airborne route [34,35].

Studies on reverse genetics-derived viruses have led to the identification of specific residues and/or gene segments that play a role in transmissibility. The presence of α2,6 SA binding in the HA and lysine at residue 627 in the PB2 protein were critical determinants of transmissibility in the 1918 H1N1 virus [36]. The Eurasian NA and M gene segments [37] and HA/NA balance [38] contributed to the transmissibility of the H1N1pdm09 virus. The HPAI H5N1 virus has not been seen to transmit from person to person in nature and is not transmissible in the ferret model. In separate studies, Imai [39] and Herfst [40] demonstrated that H5N1 viruses that became transmissible after passage in ferrets had acquired mutations in the HA that enhanced the thermal and acid stability of the protein. It is possible that the phenotype of transmissibility may be achieved by different mutations in different IAVs. 

Do host factors play a role in IAV transmission? The conventional wisdom was that only IAVs with an HA that bound α2,6 SA could transmit by the airborne route. The HAs of the 1957 and 1968 pandemic IAVs were derived from avian species and changed in their α2,3 SA binding specificity to bind α2,6 SA as they adapted to humans [41]. We used reverse genetics to convert SA binding of an H1N1pdm09 IAV from α2,6 SA to α2,3 SA and evaluated transmissibility by the airborne route in the ferret model [42]. We found that acquisition of long-chain α2,6 SA binding was required, without loss of α2,3 SA binding and that the mutation that permitted this occurred in the soft palate of the ferret, a site that was overlooked in all previous influenza research [42]. We found that the soft palate of ferrets, pigs and humans is highly enriched for long-chain α2,6 SA. This may explain why IAVs with preferential long chain α2,6 SA binding were selected in the soft palate of ferrets [42].

## 9. Additional Questions to Consider

Are novel IAV infections in humans more common now than previously? Although it is likely that the number of reported avian IAV infections in humans has increased in part due to enhanced surveillance and improved detection methods, changes in agricultural practices to large-scale farming along with attendant biosecurity issues may have altered the nature of exposure at the animal–human interface.

How do pandemics begin? Does a novel virus cause multiple abortive infections that fail to transmit from person to person before it acquires the ability to transmit by the airborne route?

What can we do to prepare? The medical, social and economic consequences of a pandemic and the certainty that another influenza pandemic will occur are so great that the public health system must prepare for the emergence of a novel IAV. The World Health Organization (WHO) recommends that all countries develop and update a pandemic preparedness plan that includes whole-of-society responsibility (https://www.who.int/influenza/preparedness/pandemic/en/). Gaps in pandemic preparedness strategies revealed during the response to the 2009 H1N1 pandemic are being addressed by policy makers. Some of the key challenges in pandemic preparedness are maintaining support for surveillance at the animal–human interface, the time it takes to manufacture and implement a specific vaccine, communications, public trust and inequities in access to health care at the global and at local levels.

Surveillance in animals and in humans who are at increased risk because of exposure at the animal–human interface is critical. Over the past two decades, the concept of One Health, that recognizes that the health of people is connected to the health of animals and the environment, has emerged and been embraced globally. One Health strategies expand collaboration and communication between the agricultural and human health sectors to achieve better public health outcomes. (https://www.who.int/features/qa/one-health/en/; http://www.oie.int/en/for-the-media/onehealth/). The WHO and the World Organization for Animal Health (OIE) work together closely to identify and characterize viruses that pose a potential pandemic threat. The WHO Global Influenza Surveillance and Response System (GISRS) reviews available data regularly, with active participation from OIE, to select candidate vaccine viruses for vaccine development and pandemic preparedness; this information is posted and updated on the WHO website (https://www.who.int/influenza/vaccines/virus/characteristics_virus_vaccines/en/).

Vaccines are the most effective approach to prevent and treat influenza. However, pandemic influenza vaccines pose several challenges [43,44,45]; currently licensed vaccines take months to manufacture and distribute and they generate highly strain-specific immunity. There are active efforts to develop universal influenza vaccines that will provide protection against a broad range of subtypes [46]. Until such vaccines are available, antiviral drugs will play a critical role in the early stages of a pandemic. 

In summary, there is a large, diverse reservoir of IAVs in nature, from which, on rare occasions, novel viruses are introduced into humans. In most instances, these cross-species incursions cause sporadic infections that range in severity from mild to severe and fatal. On four occasions in the last 100 years, a novel IAV derived from a bird or pig infected humans and spread efficiently from person to person to cause an influenza pandemic. The global impact of influenza pandemics drives scientific efforts to understand the barriers to interspecies transmission at the level of the virus and the host. It is important to elucidate how some IAVs cross the species barrier and, furthermore, why a small subset of those are able to go on to cause a pandemic. 
